# First report on the molecular detection of *Entamoeba bovis* from the endangered wild water buffalo (*Bubalus arnee*) in Nepal

**DOI:** 10.1002/vms3.697

**Published:** 2021-12-17

**Authors:** Menuka Aryal, Roshan Babu Adhikari, Prakriti Kandel, Tirth Raj Ghimire, Deegendra Khadka, Jyoti Maharjan, Kamal Prasad Gaire, Saurav Shrestha, Krishna Das Manandhar, Ram Chandra Kandel, Ram Chandra Poudel, Kishor Pandey

**Affiliations:** ^1^ Central Department of Biotechnology Tribhuvan University Kathmandu Nepal; ^2^ Third Pole Conservancy Bhaktapur Nepal; ^3^ Kathmandu University Dhulikhel Nepal; ^4^ Department of Zoology, Tri‐Chandra Multiple Campus Tribhuvan University Kathmandu Nepal; ^5^ Nepal Academy of Science and Technology Lalitpur Nepal; ^6^ Kathmandu Nepal; ^7^ Sustainable Development Initiative Centre Kathmandu Nepal; ^8^ Department of National Parks and Wildlife Conservation Ministry of Forests and Environment Kathmandu Nepal; ^9^ Central Department of Zoology Tribhuvan University Kirtipur Nepal

**Keywords:** Arna, Chitwan National Park, Entamoeba bovis, Nepal, PCR, wild water buffaloes

## Abstract

**Background:**

The Asiatic wild water buffalo (*Bubalus arnee)* is an endangered species that is conserved in the Koshi Tappu Wildlife Reserve (KTWR), Nepal, and was recently translocated to the Chitwan National Park (CNP). Gastrointestinal (GI) parasites are the cause of significant negative health and production impacts on animals worldwide.

**Methods:**

A coprological survey of GI parasites of wild water buffalo was carried out in the CNP in 2020. Fresh dung samples (n = 25) were collected from wild water buffaloes and analysed using sedimentation and flotation techniques for morphological identification of parasite cysts, oocysts and eggs.

**Results:**

Nine different GI parasites were recorded of which *Entamoeba* spp. (20 samples, 80%) were the most common. The presence of *Entamoeba* spp. was further validated using polymerase chain reaction (PCR) analysis and DNA sequencing. The PCR results were positive for all of the microscopically positive samples, and the species was identified as *Entamoeba bovis*. Three samples were sequenced and formed a cluster of *E. bovis*, which was separated from other *Entamoeba* spp. in phylogenetic analysis.

**Conclusion:**

This is the first report for molecular detection of *E. bovis* from wild water buffaloes in Nepal. Future work should focus on the prevalence of such infections in water buffaloes in forest environments.

## Graphical abstract

1. The Asiatic wild water buffalo is an endangered species that are present only two areas in Nepal.

2. Microscopic examination revealed 96% prevalence rate with four protozoan and five helminths’ parasites detected in wild water buffalo.

3. PCR and sequencing revealed the presence of highly prevalent species as Entamoeba bovis.

4. This is the first molecular report of *E. bovis* in Nepalese wild water buffaloes.

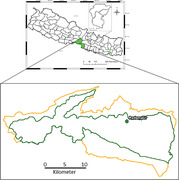

## 1 INTRODUCTION

Gastrointestinal (GI) parasitic infection is a major problem in livestock and wildlife management, including wild water buffaloes (Bista et al., 2018). GI parasites may induce mild to severe pathological consequences, such as diarrhoea, loss of appetite, weight loss, haemorrhagic colitis, anaemia and infertility, thus contributing to higher morbidity and mortality in the animals (Houszka, 2001; Otranto, 2011). The protozoan *Entamoeba* spp. are critical zoonotic parasites that infect both animals and humans (Feng et al., 2018; Tachibana et al., 2013, 2007; Win et al., 2020), and are transmitted among hosts through the ingestion of cysts in contaminated food, water or faeces (Matsubayashi et al., 2018). Major pathological illnesses caused by *Entamoeba* spp. infection are diarrhoea and dysentery, as well as damage to the liver, brain and other organs of the host (Ngui et al., 2012). Several *Entamoeba* spp. are found in mammals, but the pathogenesis is caused mainly by *Entamoeba histolytica*. It has been reported that nearly 100,000 people die of amoebiasis worldwide every year (Stensvold et al., 2010). Globally, it is the second most important protozoan parasite that causes fatality after malaria (Delialioglu et al., 2008). *Entamoeba bovis* and similar species inhabit the rumen of ruminant mammals and are morphologically identical to *E. histolytica* (Stensvold et al., 2010). *E. bovis* has been reported from different hosts, including cattle, goat, sheep, deer, reindeer, gnu and bay duiker (Jacob et al., 2016; Matsubayashi et al., 2018; Ren et al., 2021; Stensvold et al., 2011). To date, nine countries (Australia, China, Costa Rica, Iceland, Japan, Libya, Sweden, Uganda and United Kingdom) reported cases of *E. bovis* (Al‐Habsi et al., 2017; Matsubayashi et al., 2018; Ren et al., 2021).

Traditionally, light microscopy and morphological identification are used to diagnose parasites within faecal samples, including *Entamoeba* spp. (Shrestha et al., 2018). However, such microscopic diagnostics are less effective because of a lack of sensitivity and specificity (Win et al., 2020). It is difficult and challenging for positive identification because of similar morphological characters among *Entamoeba* spp. and a lack of adequate knowledge on their host‐specificity (Stensvold et al., 2011) In this regard, PCR‐based diagnosis with high sensitivity and specificity is effective in detecting and identifying GI parasites, but this technique is rarely used in Nepal (Singh et al., 2009).

The wild water buffalo, a large bovidae family ungulate, is a herd‐loving mega‐herbivore. The Asiatic wild water buffalo, also known as Arna (*Bubalus arnee*), is listed as a protected species by the National Parks and Wildlife Conservation Act, 1973 (GoN, 1973). This species is categorised as Endangered on the IUCN Red List and Appendix III of the Convention on International Trade in Endangered Species (Kaul et al., 2019). Historically, wild water buffalo populations were distributed across southeast Asia from Mesopotamia to Indo‐China (Fischer, 1976). At present, as the climate becomes drier, they are restricted to small pockets in scattered populations in Nepal, India, Bhutan and Myanmar. The habitat of these animals is shrinking substantially (Khatri et al., 2012; Kandel et al., 2019a).

Wild water buffaloes existed in the Chitwan National Park (CNP) until the early 1960s, and it is assumed that they became extinct due to disease transferred by local livestock and hunting (Seidensticker, 1975). Currently, a population of 498 individuals exists in the Koshi Tappu Wildlife Reserve (KTWR), which was established solely to protect the remaining population (<60 individuals during establishment) (Kandel et al., 2019a). The KTWR has abundant habitats suitable for wild water buffaloes, such as grassland and swampy areas. However, these protected herds are at continuous risk of extinction due to extreme flooding during the monsoon, and higher susceptibility of disease transmission from domestic buffaloes (Khatri et al., 2012). Therefore, several researchers recommended translocating some buffalo from the KTWR into either the CNP or the Bardia National Park (Aryal et al., 2011; Heinen, 1993) for long‐term conservation. The translocation, introduction and reintroduction of species to the areas within their former range, or the areas considered appropriate for their survival and persistence, are proposals that are widely promoted in conservation biology (Osborne and Seddon, 2012; Thapa et al., 2020). Following three decades of recommendations by various experts, the translocation of 18 wild water buffaloes to the CNP took place in January–February 2017 (Kandel et al., 2019a; Shah et al., 2017). Some of the recently translocated wild water buffaloes died due to parasitic diseases, tiger attacks and the consequences of a severe flood (DNPWC, 2020).

A previous study detected ungulate malaria parasites in wild water buffaloes for the first time from Nepal (Kandel et al., 2019b). Thus, the current coprological survey has been conducted to assess the health of wild water buffaloes in the new habitat through a study of the presence of GI parasites. In this study, *E. bovis* was identified as the most dominant intestinal parasite as observed by microscopic methods, PCR and DNA sequencing.

## 2 METHODOLOGY

### 2.1 Study area

**FIGURE 1:**
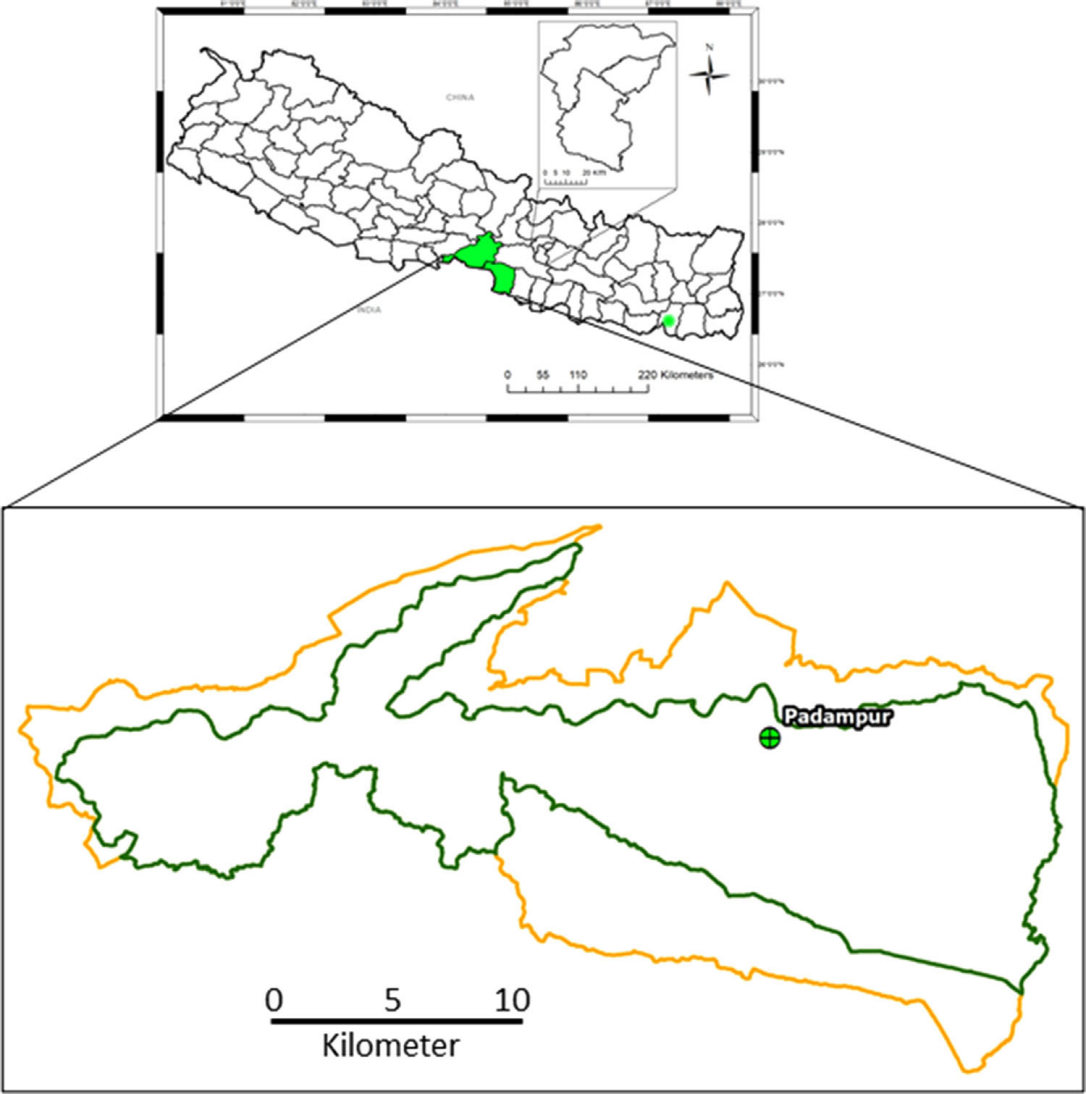
Map of Nepal showing the location of sample collection. Green asterisk denotes the Koshi Tappu Wildlife Reserve

In 1973, the CNP was declared the first protected area of Nepal and was included in the World Heritage Site in 1984 (Bhuju et al., 2007). The park is located in southern central Terai of Nepal and has an area of 953 km^2^ (Figure 1). It is home to 70 species of mammals, 49 species of reptiles and amphibians, 541 species of birds and 120 species of fish (CNP, 2021). Eighteen translocated wild water buffaloes were kept in an enclosure of 30 hectares in the Padampur area of the CNP, and the enclosure is protected by a solar fence. This translocated population is monitored daily to record their condition.

### 2.2 Sample collection

Wild buffaloes are herding social animals, and usually rest in a group at night at a certain locality. Calves always remain close to their mothers. We followed each individual buffalo to their resting place and collected fresh dung nearest to an individual for accurate sampling. We also sampled additional fresh dung to represent the few buffaloes that wander alone and live distant from the herd. The study team was accompanied by the Assistant Conservation Officer and two staff members who regularly supervise the enclosure. A total of 25 fresh faecal samples (dung) were non‐invasively collected in September 2020 from the water buffaloes. Before collecting the faecal samples, each specimen was carefully examined for the blood and mucus contained. Then, about 10 gm was collected in a plastic vial with the help of a sterile spatula. The collected samples were mixed in 2.5% weight/volume (w/v) potassium dichromate solution, transported to the laboratory and stored at 4°C until microscopic and molecular examination.

### 2.3 Microscopic examination

Flotation and sedimentation methods were used for the microscopic examination of the faecal samples based on established protocols (Adhikari et al., 2020). In the sedimentation technique, about 2 gm of the faecal sample was mixed in 13 mL of normal saline. The samples were filtered using a tea strainer into a 15 mL conical centrifuge tube and centrifuged at 1200 rpm for 5 min. The supernatant was discarded; and one to two drops of sediment were transferred to a glass slide for microscopic observation using Gram's iodine stain. For the flotation technique, the sediment was put in a 12 mL saturated salt solution (45% w/v) in the centrifuge tube and recentrifuged (1200 rpm × 5 min). In the next step, salt solution was added drop by drop to completely fill the tube, and a coverslip was placed at the mouth of the tube so that the coverslip would touch the flotation media. After 10 min, the coverslip was carefully removed and placed on the glass slide. Then, the samples were observed under a light microscope (Optika Microscopes Italy, B‐383PLi) at magnifications of 100× and 400×. Microscopic images of the parasites were captured by a camera (SXView 2.2.0.172 Beta [6 November 2014) Copyright (C) 2013–2014). Finally, the micrometry of the reported parasitic bodies (eggs/oocysts/cysts) was assessed by ImageJ 1.51k (National Institute of Health, USA). The cysts, oocysts and eggs of the intestinal parasites were identified morphologically based on the literature (Adhikari & Ghimire, 2021; Soulsby, 2012; Zajac et al., 2013).

### 2.4 DNA extraction, PCR and sequencing

Genomic DNA was extracted from each faecal sample using a QIAamp DNA Stool Mini Kit (Qiagen, Hilden, Germany). To obtain DNA in high yield and good quality, prior to purification, the preserved faecal samples in 2.5% potassium dichromate solution (w/v) were subjected to freeze‐thaw‐boiling and bead beating processes. Briefly, samples were mixed in 0.9% NaCl, sieved using a plastic tea strainer and centrifuged (1500 rpm × 5 min). The pellet was washed twice with phosphate buffered saline (PBS) (1500 rpm × 5 min). Then, 200 μL PBS was added and 666 μL of aliquot was transferred to a fast prep 2 mL Lysing Matrix tube (Matrix A, CA, USA). The mixture was frozen in liquid nitrogen for 2–3 min or kept at –20°C for 30 min, followed by a thaw‐boiling phase in a preheated heating block at 100°C for 10 min. The sample was then subjected to bead beating in a tissue lyser (MP Biomedicals FastPrep‐24 5G) at 6 m/s for 65 s. After lysis, the DNA was extracted following the manufacturer's protocol with modification in the heating steps (70°C for 30 min). DNA was eluted and stored at −20°C until PCR analysis.

For the identification of *Entamoeba* spp., the described primers, JVF and DSPR2, were used targeting the 18S gene (Ren et al., 2021). PCR amplification was performed in a 10 μL reaction with 5 μl 1XTaqR Green Master mix, 0.4 μL of 10 μM of each primer, 1 μL of DNA template and nuclease‐free water. The PCR programme was performed as follows: 5 min at 95°C, 40 cycles for 30 s at 95°C, 30 s at 57°C and 30 s at 72°C, and then 2 min at 72°C for the final extension. PCR products were analysed by separation on 2% agarose gel electrophoresis at 100 V for 30 min, stained with ethidium bromide, and visualised in a Gel Doc system (Syngene InGenius, Cambridge, UK).

Primers contaminating the PCR products were removed using 2 μL of Sap‐Exo (Jena Bioscience, Jena, Germany) for 5 μL of PCR product and incubated at 37°C for 10 min followed by 80°C for 10 min in a thermocycler. The purified products were applied to a sequencing reaction using a BigDyeTM Terminator v3.1 Cycle Sequencing Kit (Applied Biosystems, CA) according to the manufacturer's protocol using forward and reverse primers. These sequencing reactions were purified using a Big Dye XterminatorTM Kit (Applied Biosystems) and analysed in an automated 3500XL Genetic Analyzer (Applied Biosystems). Raw DNA sequence data were assembled and aligned using Sequencer 5.0 (Thompson et al., 1997), and DNA contigs were generated using forward and reverse sequences. Consensus sequences thus obtained were aligned with published *Entamoeba* spp. using Bio edit v7.2.5 (Hall, 1999).

### 2.5 Data analysis

Microscopic observation data of each sample were tabulated in Microsoft Excel, and GraphPad software was used to plot the data. The DNA sequence matrix thus formed was aligned using the ClustalW programme (Thompson et al., 1994), and then exported to MEGA V7 (Kumar et al., 2016) for phylogenetic analysis. A phylogenetic tree was constructed using a Neighbor‐Joining algorithm based on 10,000 bootstraps.

## 3 RESULTS

**FIGURE 2:**
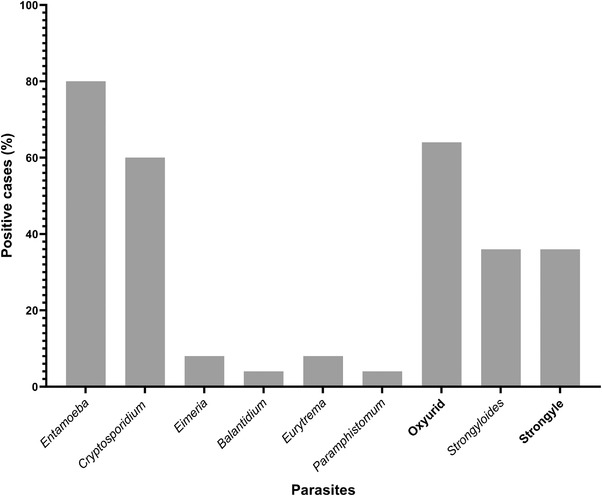
The proportions of gastrointestinal parasite infections (n = 24) in wild water buffaloes at Chitwan National Park

**FIGURE 3:**
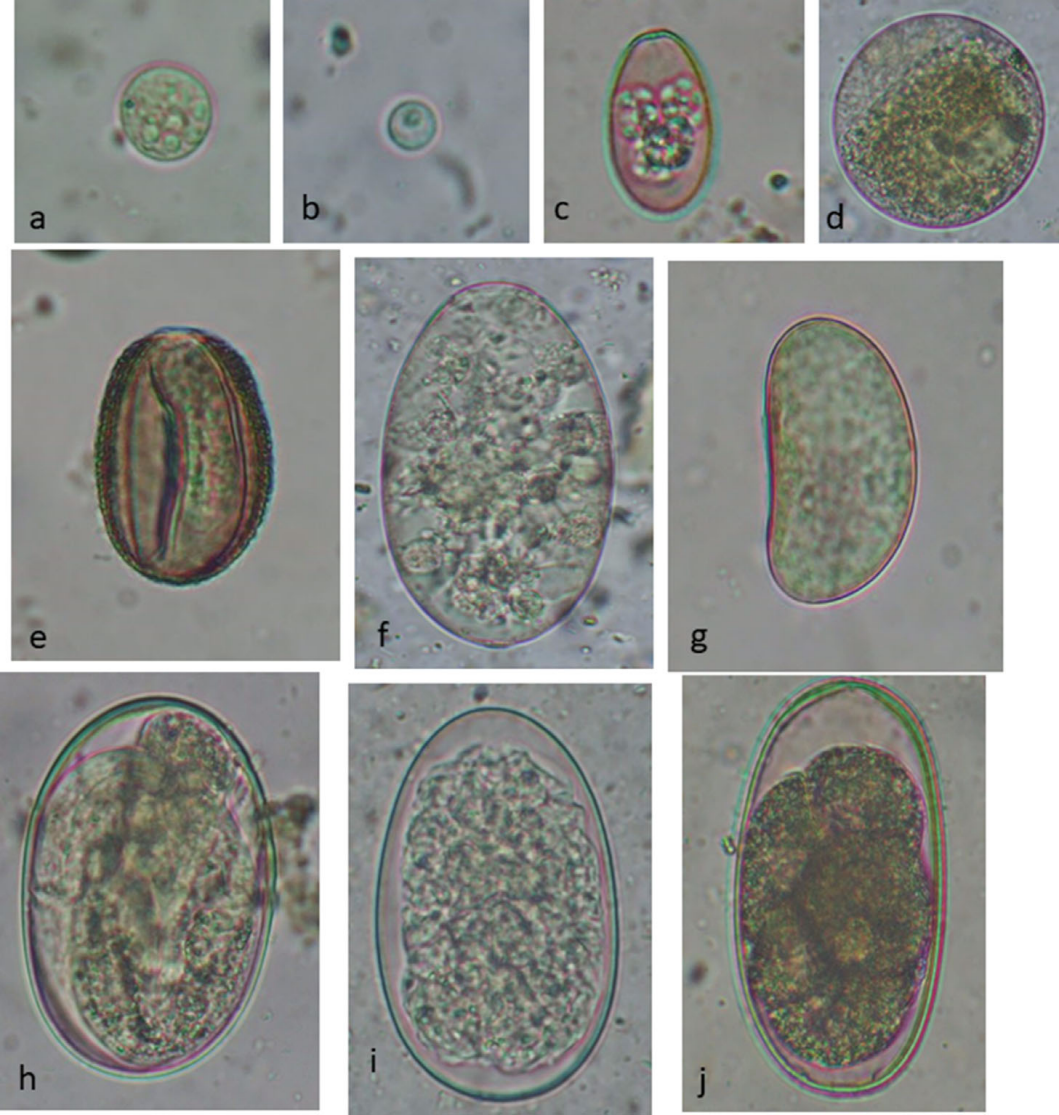
Gastrointestinal parasites detected in the wild water buffaloes. Microscopic images of the parasites detected after sedimentation and flotation techniques, staining with Gram's iodine and visualisation with the 40× objective lens magnification of a compound microscope. (a) Cyst of *Entamoeba* sp. (13 × 12 μm). (b) Oocyst of *Cryptosporidium* sp. (6 × 6 μm). (c) Oocyst of *Eimeria* sp. (26 × 18 μm). (d) Cyst of *Balantidium coli* (71 × 70 μm). (e) Egg of *Eurytrema* sp. (37 × 26 μm). (f) Egg of *Paramphistomum* sp. (121 × 53 μm). (g) Egg of Oxyurid sp. (40 × 24 μm). (h) Egg of *Strongyloides* sp. (75 × 49 μm). (i) Egg of Strongyle sp. type 1 (88 × 54 μm). (j) Egg of Strongyle sp. type 2 (78 × 43 μm)

Out of 25 faecal samples, 24 (96%) were positive for GI parasitic species from wild water buffaloes protected at the Padampur enclosure of the CNP. Among the studied samples, 22 (88%) were positive for protozoan parasites, and 21 (84%) were positive for helminth parasites (Figure 2). Nine different GI parasites were detected by microscopic examination (Figure 3); including four protozoa (*Entamoeba*, *Eimeria*, *Balantidium coli* and *Cryptosporidium* [Figure 3]), two trematodes (*Eurytrema* and *Paramphistomum*) and three nematodes (Oxyruid*, Strongyloides* and Strongyle [Figure 2]). Only one faecal sample contained a single parasite (*Entamoeba*) and the remaining 23 samples were infected with multiple parasite infections. Among the detected parasites, *Entamoeba* was the most common and the cysts detected were morphologically similar. Double infections were present in eight samples (32%) followed by triple infections in six samples (24%), quadruple in five samples (20%), quintuple in two samples (8%) and sextuple in two samples (8%).

**FIGURE 4:**
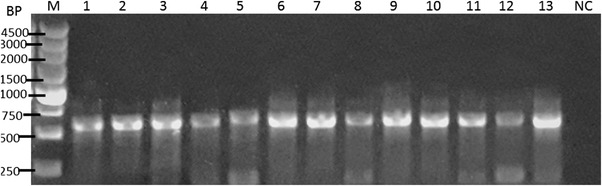
Agarose gel electrophoresis of PCR‐amplified products using *Entamoeba bovis*–specific primers. M, DNA ladder; lane 1–13, samples from wild water buffaloes; NC, negative control

**FIGURE 5:**
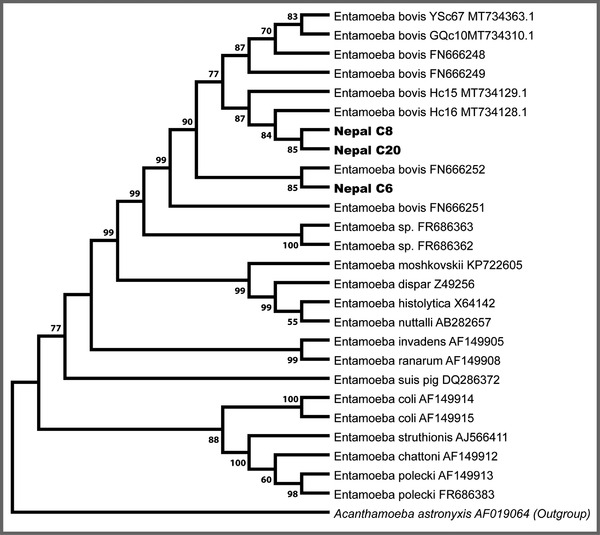
Phylogenetic tree based on the partial 18S rRNA sequences of representative *Entamoeba* members constructed using the maximum likelihood (ML) method. The GenBank accession numbers of all sequences are indicated after their taxon names. New sequences are indicated in the bold text. The scale bar represents 0.05 substitutions per nucleotide

We used PCR analysis for the differentiation of *Entamoeba* spp. All positive faecal samples (n = 20) for *Entamoeba* in microscopic examination were subjected to DNA extraction, and then PCR was performed using species‐specific primers. PCR analysis showed the PCR product of approximately 550 bp in 2% agarose gel during electrophoresis (Figure 4). Three PCR positive samples were sequenced using both forward and reverse primers and were included in phylogenetic analysis. Phylogenetic relationships between the *E. bovis* identified in this study and reference accessions downloaded from GenBank were studied through multiple sequence alignment. The results showed that *E. bovis* has the closest evolutionary relationship with *E. bovis* from yak, which together forms a monophyletic branch (Figure 5).

## 4 DISCUSSION

To the best of our knowledge, the current study is the first work to report *E. bovis* in the faecal samples of the wild water buffalo from Nepal. Few reports are available on the identification of parasites, the role of diseases on health and diagnostic techniques for water buffalo. A parasitological study is needed to identify the common parasites and understand the mode of infection, potential risks of transmission between species and their impact on health and reproduction. The present study provides baseline information on the parasitic prevalence in the translocated populations of wild water buffaloes and thus may be useful to formulate appropriate control strategies in these hosts.

The overall prevalence of intestinal parasites in wild water buffaloes was 96%, with 88% protozoa and 84% helminths. These rates were higher than reported by another study in which the prevalence rate was 48% (n = 160) and a lower divergence of intestinal parasites with protozoa (35%), nematodes (39%) and trematode (23%) was recorded (Gupta, 2017). Similarly, mixed infection (90%) was also found to be higher in our study. In a study from Sri Lanka, 75%, 36% and 64% of these hosts were infected with *Entamoeba* spp., *Balantidium coli* and *Isospora* spp., respectively (Sepalage & Rajakaruna, 2020). Similarly, the presence of *Cryptosporidium* sp. had been suggested in the KTWR buffalo population (Chalise, 2014), indicating that protozoan parasites are common in wild water buffaloes. The buffaloes in the KTWR are free ranging, compared to the buffaloes sampled in this study that were within a fenced enclosure and a relatively controlled environment. Thus, grazing in marshy lands and contact with risk factors such as the presence of metacercaria larva and host plants of trematodes are usually lacking. However, the continuous use of the same pasture and resources increases the risk of reinfection or transmission of similar parasite species within the population (Kumar et al., 2014). Consequently, only *Eurytrema* and *Paramphistomum* sp. were detected in the faecal samples in the current study.

In the current study, both microscopic and PCR analyses showed that 80% of the wild water buffaloes were infected with *E. bovis*. A study done in China showed that 100% of cattle and >90% of yak, goat and sheep were infected with *E. bovis* (Ren et al., 2021). *E. bovis* infection was detected in 80% of cattle and 60% of goats in a study conducted in Uganda (Nolan et al., 2017).

In the present study, PCR analysis of the faecal samples collected from the study area corresponded with the microscopic examinations, and DNA sequencing confirmed *Entamoeba* to the species level. Specifically, all successfully amplified and sequenced samples corresponded to *E. bovis*, and clustered with reported *E. bovis* sequences (Matsubayashi et al., 2018; Nolan et al., 2017).

The limitation of this study was the use of a smaller sample size, although it covered all individuals translocated to the CNP. Additional surveys of wild water buffaloes with a high sample number in the KTWR are needed to clarify the prevalence of *Entamoeba* parasites. Thus far, there is no report about the pathogenicity of *E. bovis* in animals (Matsubayashi et al., 2018). Little is known about the intensification of GI symptoms by mixed parasite infection of ruminant *Entamoeba* spp. with other intestinal parasites (Matsubayashi et al., 2018; Ren et al., 2021). Therefore, further study is needed to determine the pathologic consequences of *Entamoeba* in mixed‐infection situations.

## 5 CONCLUSIONS

The high prevalence of intestinal parasite infections in the translocated wild water buffalo population poses a significant threat in the CNP. This study provides baseline information on intestinal parasites including *E. bovis* for the first time in Nepalese wild water buffaloes. The outcomes of this research work can be applied to the control and management of intestinal parasites in wild water buffaloes. GI parasites could be control by applying a regular deworming and amebicide programme to the managed animals. Control of these parasites should be integrated with the extensive conservation efforts made by the government, local communities and conservation groups to protect the last remaining population of the endangered wild water buffaloes in Nepal.

## ETHICAL STATEMENTS

The study was approved by the Department of National Parks and Wildlife Conservation, Kathmandu, Nepal.

## CONSENT OF PUBLICATION

Not required.

## AVAILABILITY OF DATA AND MATERIALS

The dataset supporting the conclusions of this article are included within this article.

## CONFLICTS OF INTEREST

There are no conflicts of interest for this work.

## AUTHOR CONTRIBUTIONS

RCK, RCP and KP contributed to the conception of the study. MA, RBP, PK, TRG, RCK, RCP and KP designed the study. PK, SS, RCK, RCP and KP carried out the field visit; MA, RBP, PK and TRG carried out the microscopic examinations; and MA, PK, RCP and KP carried out the molecular work. DK, JM, KPG, SS, RCP and KP analysed and interpreted the data. TRG, DK, KPG, SS, RCP and KP drafted the manuscript. All the authors have approved the submitted version of the manuscript.

### PEER REVIEW

The peer review history for this article is available at https://publons.com/publon/10.1002/vms3.697
